# Cardiac hypertrophy associated with myeloproliferative neoplasms in JAK2V617F transgenic mice

**DOI:** 10.1186/1756-8722-7-25

**Published:** 2014-03-19

**Authors:** Kaiyao Shi, Wanke Zhao, Yun Chen, Wanting Tina Ho, Ping Yang, Zhizhuang Joe Zhao

**Affiliations:** 1 Department of Cardiology, China-Japan Union Hospital of Jilin University, Changchun 130021, China; 2 Department of Pathology, University of Oklahoma Health Sciences Center, Oklahoma City, Oklahoma 73104, USA

**Keywords:** MPN, JAK2V617F, Hematological malignancies, Fibrosis, Cardiomegaly, Cardiac hypertrophy

## Abstract

**Background:**

Myeloproliferative neoplasms (MPNs) are blood malignancies manifested in increased production of red blood cells, white blood cells, and/or platelets. A major molecular lesion associated with the diseases is JAK2V617F, an activation mutation form of tyrosine kinase JAK2. Cardiovascular events represent the leading cause of morbidity and mortality associated MPNs, but the underlying mechanism is not well understood.

**Methods:**

Previously, we generated JAK2V617F transgenic mice which displayed MPN-like phenotypes. In the present study, we further characterized these mice by analyzing the time course of MPN phenotype development and associated cardiac abnormalities. We performed detailed histochemical staining of cardiac sections.

**Results:**

JAK2V617F transgenic mice developed cardiomegaly as a subsequent event of increased blood cell production during the course of MPN phenotype development. The cardiomegaly is manifested in increased ventricular wall thickness and enlarged cardiomyocytes. Trichrome and reticulin staining revealed extensive collagen fibrosis in the heart of JAK2V617F transgenic mice. Thrombosis in the coronary artery and inflammatory cell infiltration into cardiac muscle were also observed in JAK2V617F transgenic mice, and the latter event was accompanied by fibrosis.

**Conclusion:**

JAK2V617F-induced blood disorders have a major impact on heart function and lead to cardiac hypertrophy. JAK2V617F transgenic mice represent an excellent model system to study both hematological malignancies and cardiovascular diseases.

## Background

Myeloproliferative neoplasms (MPNs) are chronic hematopoietic malignancies characterized by abnormal amplification of one or more myeloid lineages [1-3]. These diseases include polycythemia vera (PV), essential thrombocythemia (ET), and primary myelofibrosis (PMF) [4,5]. PV patients have increased production of all three types of blood cells, whereas ET patients mainly show elevations of platelets. Patients with PMF develop fibrous (scar-like) tissues in the bone marrow as a result of abnormal myeloid proliferation. MPNs mainly affect older people with an average onset of 55 years. So far, there is no effective cure for the diseases [6-9]. Complications associated with MPNs include thrombosis, hemorrhage, heart attacks, strokes, myeloid metaplasia, and acute leukemia. Strokes and heart attacks caused by these diseases are usually fatal. In fact, cardiovascular events represent the leading cause of morbidity and mortality in the course of PV and ET [10-12]. However, how the blood abnormality associated with MPNs leads to cardiac changes is not well understood.

JAK2V617F, a mutant form of tyrosine kinase JAK2, represents a major molecular defect in MPNs [13-17]. It is found in over 95% of PV and over 50% of ET and PMF cases [1-3,18]. In early studies, we have generated JAK2V617F transgenic mice by using the *vav-1* promoter which drives transgene expressions in the hematopoietic system. JAK2V617F transgenic mice display MPN-like phenotypes with increased levels of red blood cells, platelets, and white blood cells [19]. These mice thus represent a model system to study MPNs and associated complications. In the present study, we investigated cardiac changes and remodeling during the course of MPN phenotype development in these mice. Our data demonstrate that JAK2V617F transgenic mice display cardiac hypertrophy manifest in increased ventricular wall thickness, enlarged cardiomyocytes, and formation of collagen fibrosis in the heart. Our study thus links a chronic blood malignancy directly to heart diseases.

## Results

### JAK2V617F transgenic mice displayed cardiomegaly

**Figure 1:**
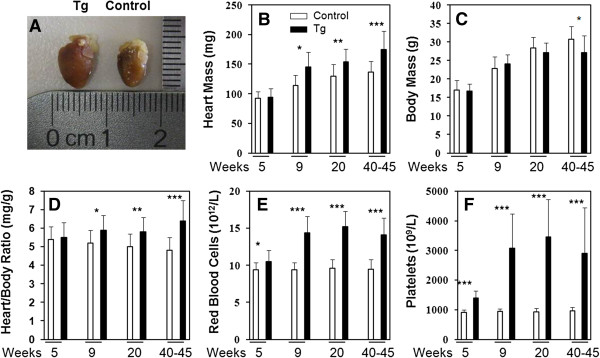
**JAK2V617F mice develop cardiomegaly associated with increased blood cell counts.** Photos of hearts from typical control and JAK2V617F transgenic mice at age of 40–45 weeks are shown **(A)**. Bar graphs represent average heart mass **(B)**, body mass **(C)**, heart/body ratio **(D)**, red blood cell counts **(E)**, and platelet counts **(F)** of control (open bar) and JAK2V617F transgenic (closed bar) mice at indicated ages. Error bars denote standard deviation (n ≥10). * P <0.05, **P < 0.01, ***P < 0.001.

Previously, we have generated JAK2V617F transgenic mice that displayed MPN-like phenotypes with elevated platelet and red blood cell counts [19]. This provides us with a model system to study MPNs and complications associated with MPNs. We employed homozygous line A JAK2V617F transgenic mice in this study to demonstrate the effects of JAK2V617F-induced MPN phenotypes on heart function. Initially, we noticed apparent cardiomegaly, namely, heart enlargement, in these mice as illustrated in Figure 1A. Further detailed analyses of more than 10 mice in each group revealed that cardiomegaly was seen in JAK2V617F transgenic mice at 9 weeks of age (P < 0.05) and became more remarkable as the mice aged (Figure 1B). At the age of 40–45 weeks, hearts from JAK2V617F transgenic mice were nearly 30% heavier than those of control mice on average (P < 0.001). When the body weight is taken into consideration, the difference is more prominent because at 40–45 weeks, the transgenic mice weighed significantly less than the control mice (Figure 1C and D). Note that at a younger age of 5 weeks, there is no difference in the heart mass although the transgenic mice clearly showed MPN phenotypes as indicated by elevated levels of red blood cells and platelets (Figure 1E and F). The data suggest that the onset of MPN phenotypes preceded the development of cardiomegaly in JAK2V617F transgenic mice. The cardiomegaly is apparently not from birth but rather developed as a consequence of MPNs.

**Figure 2:**
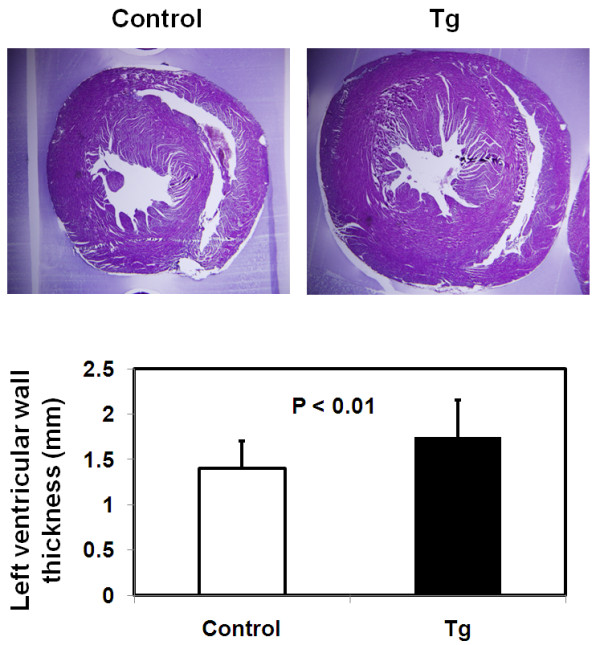
**Cardiomegaly in JAK2V617F mice is manifested in increased ventricular wall thickness.** The top panel shows representative H&E staining of middle transverse cardiac sections from 40–45 week-old control and JAK2V617F transgenic mice (magnification, ×1.6). Data in the bar graph represents mean ± SD (n ≥10). Note that the left ventricular wall thickness was significantly increased in JAK2V617F transgenic mice (P < 0.01).

**Figure 3:**
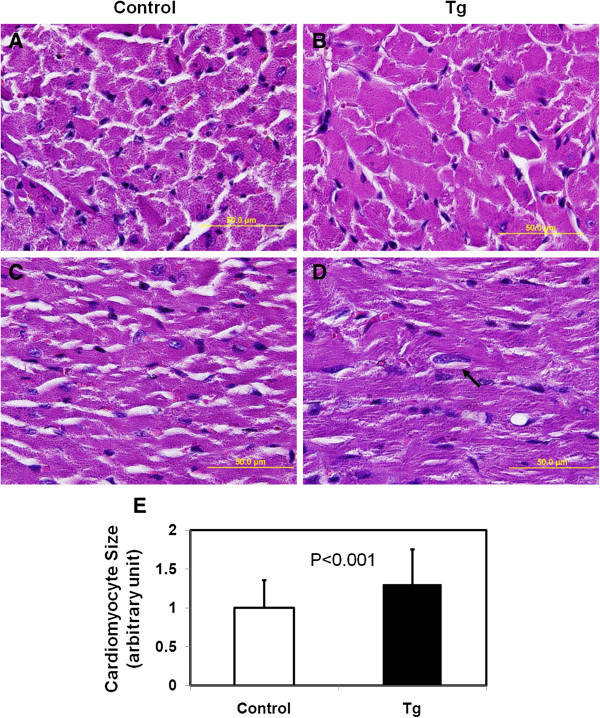
**The size of cardiomyocytes is significantly increased in JAK2V617F transgenic mice.** Representative histology of H&E-stained cardiac sections corresponding to the left ventricle free wall of hearts from 40–45 week-old control **(A and C)** and JAK2V617F **(B and D)** (magnification, ×100). A and B show myofibers running parallel to the apical-basal axis, and B and D show myofibers running circumferentially. Note that an enlarged nucleus is seen in a hypertrophic myocyte (pointed by an arrow in **D**). Data in the bar graph **(E)** represent cross-sectional area measurements of at least 20 cardiomyocytes per animal shown in A and B from 10 control and 10 JAK2V617F mice. Error bars denote SD (n ≥200). P < 0.001.

We employed mice of 40–45 weeks to characterize the cardiomegaly further. Microscopic examinations of hematoxylin and eosin (H&E)-stained cardiac sections under low magnification revealed significant thickening of the left ventricular wall in the JAK2V617F transgenic mice (P < 0.01, Figure 2). Further exanimation under high magnification demonstrated enlarged cardiomyocytes (P < 0.001, Figure 3). On average, the cells from the transgenic mice were 30% bigger in area than the control. In addition, enlarged nuclei were seen in some hypertrophic cardiomyocytes (Figure 3D), suggesting an increase in DNA ploidy due to DNA replication in the absence of cell division. Therefore, the cardiomegaly observed in JAK2V617F transgenic mice can be partly attributed to thickening of the left ventricular wall and enlargement of cardiomyocytes.

### JAK2V617F transgenic mice developed fibrosis in the heart

**Figure 4:**
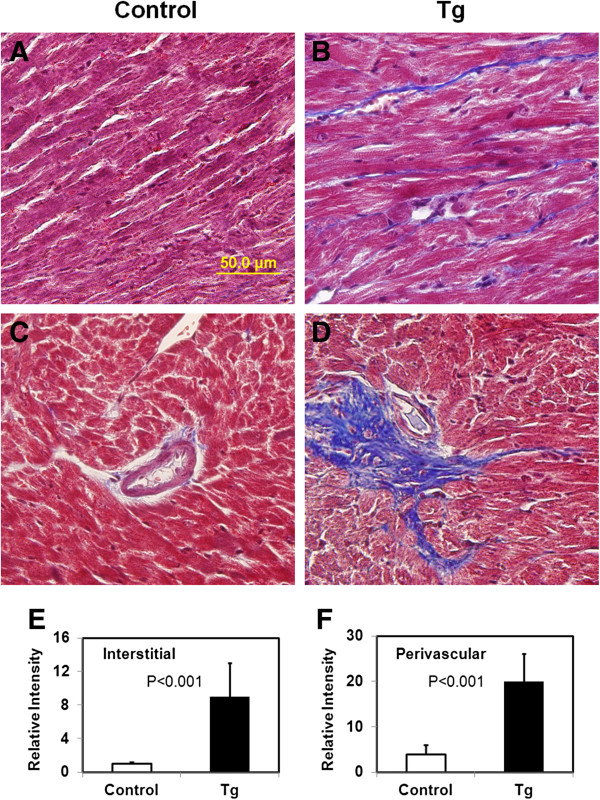
**Masson’s Trichrome stain reveals collagen fibrosis in hearts from JAK2V617F transgenic mice.** Representative trichrome stains of heart sections from 40–45 week-old control **(A and C)** and JAK2V617F transgenic **(B and D)** mice are shown (magnification, ×40). Note the extensive interstitial **(B)** and perivascular **(D)** fibrosis represented by the blue stains. Data in the bar graphs represent quantitative measurements of interstitial **(E)** and perivascular **(F)** trichrome staining intensity of at least 5 randomly selected areas from each of 10 control and 10 JAK2V617F transgenic mice. Error bars denote SD (n ≥50). P < 0.001.

To define the cellular and molecular mechanism underlying the development of cardiomegaly in JAK2V617F transgenic mice, we performed Masson’s trichrome staining, a commonly used method to detect collagen fibers. As indicated by the bright blue staining, prominent collagen fibrosis was observed in the left ventricle of transgenic mouse hearts (Figure 4). The fibrosis occurred in both interstitial and perivascular regions (Figure 4B and D, respectively). In contrast, trichrome staining in the correspondent regions of control mouse hearts was marginal (Figure 4A and C).

**Figure 5:**
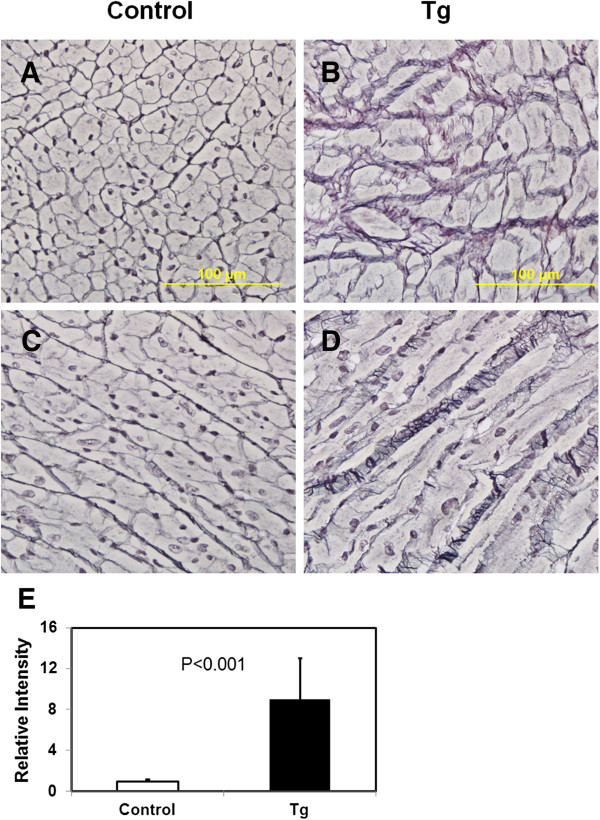
**Reticulin fibrosis is significantly increased in hearts from JAK2V617F transgenic mice.** Representative reticulin stains of heart sections from 40–45 week-old control **(A and C)** and JAK2V617F transgenic **(B and D)** mice are shown (magnification, ×40). Note that interstitial reticulin fibrosis is much more intensive in JAK2V617F transgenic mice in comparison with control mice. Data in the bar graph **(E)** represent quantitative measurements of interstitial reticulin staining intensity of at least 5 randomly selected segments from each of 10 control and 10 JAK2V617F transgenic mice. Error bars denote SD (n ≥50). P < 0.001.

Since the Masson’s trichrome staining mainly detects type I collagen, we further employed reticulin staining to investigate type III collagen. As shown in Figure 5, very intensive and thick interstitial reticulin fibers were observed with the transgenic mice. Reticulin staining in the cell-cell junction of control mouse hearts was also apparent but were much thinner. The data thus demonstrated a much higher amount of type III collagen in the heart of JAK2V617F transgenic mice.

The most abundant extracellular matrix proteins in the heart are collagens, particularly the collagen type I and type III phenotypes [20,21]. Our study thus revealed substantial accumulation of collagen fibrosis in the heart of JAK2V617F transgenic mice. This contributes to major cardiac remodeling and may significantly affect the heart function.

### Coronary artery thrombosis and inflammatory cell infiltration were found in the heart of JAK2V617F transgenic mice

**Figure 6:**
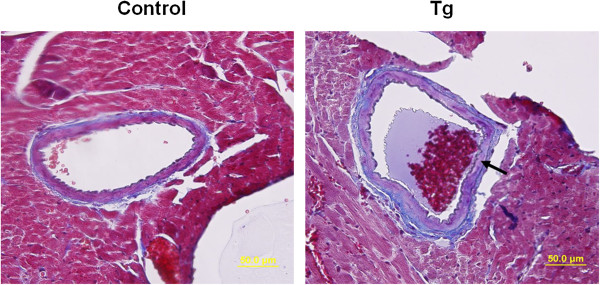
**Thrombosis occurs in the coronary artery of JAK2V617F transgenic mice.** Trichrome stains of cardiac sections demonstrate a typical thrombus in the coronary artery of 40–45 week-old JAK2V617F transgenic mice.

**Figure 7:**
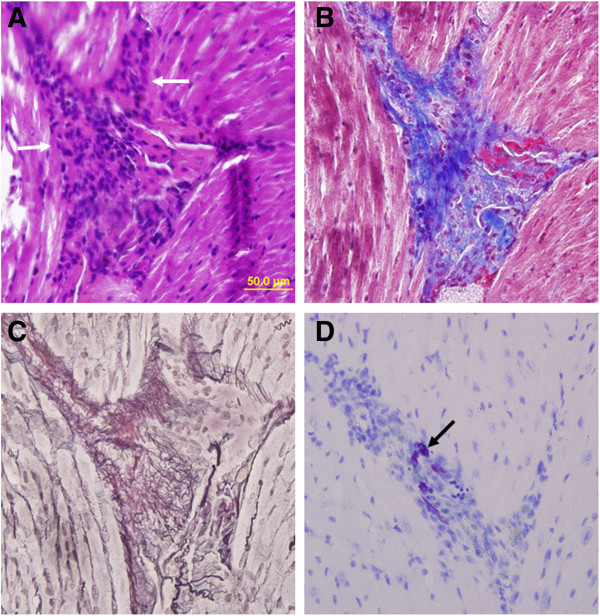
**Inflammatory cell infiltration occurs in JAK2V617F transgenic mouse hearts and is accompanied by fibrosis.** Consecutive sections of the heart from a 40–45 week-old JAK2V617F transgenic mouse were subjected to H&E **(A)**, Masson’s trichrome **(B)**, reticulin **(C)**, and toluidine blue staining **(D)** (magnification, ×40; bar = 50 um). White arrows indicate inflammatory cells, and a black arrow points to mast cells.

We further examined at least 5 selected H&E-stained cardiac sections from each of 20 control and 20 JAK2V617F transgenic mice of 40–70 weeks of age. We found occurrence of thrombosis in the coronary artery of 4 transgenic but not a single control mice (Figure 6). This suggests that some of the transgenic mice may have suffered from heart attack. Coronary artery thrombosis typically causes acute myocardial infarction. This can further deteriorate cardiac hypertrophy. We also noticed that cardiac sections from 5 JAK2V617F transgenic mice but not at all from control mice had infiltration of inflammatory cells as demonstrated in Figure 7. Trichrome and reticulin staining revealed collagen fibrosis in the affected regions. Interestingly, toluidine blue staining showed that mast cells were among the infiltrated inflammatory cells. Mast cells has increasingly been recognized as effector cells in many cardiovascular diseases, and many mast-cell-derived proteases have detrimental effects on blood vessel structure while mast cell-derived cytokines and chemokines can promote vascular inflammation [22,23].

### Real time PCR analyses revealed a minimal expression of transgenic JAK2V167F in the mouse heart

The cardiac hypertrophy observed in JAK2V617F mice is likely a consequence of MPN phenotype. However, there is a possibility that the JAK2V617F expression may directly affect cardiomyocytes. In fact, an earlier study revealed the presence of JAK2V617F in cardiomyocytes from a patient with MPNs and hypertrophic cardiomyopathy [24]. To determine the expression of endogenous Jak2 and transgenic JAK2V617F in the heart, we performed real time PCR analysis with specific primers as described previously [19]. Expression of these genes in hematopoietic tissues including bone marrow, peripheral blood, and spleen were included for comparison. Despite containing 26 copies of the JAK2V617F transgenic gene [19], mRNA transcripts of JAK2V617F were nearly 10-fold below the expression level of endogenous mouse Jak2 in the hematopoietic tissues. Mouse Jak2 was expressed at a substantial level in the heart, which is consistent with the crucial role of JAK2 in cardiomyogenesis [25]. However, expression of transgenic JAK2V617F in the heart was much lower, about 200-fold below the level of mouse Jak2 in the heart or of JAKV617F in the hematopoietic tissues. This low-level JAK2V617F expression may be mainly attributable to the presence of residual blood cells in the cardiac blood vessels. The data thus demonstrate a minimal expression of JAK2V617F transgene in mouse heart cells. Although we cannot totally rule out the contribution of JAK2V617F expression in cardiomyocytes to the development of cardiac hypertrophy phenotypes, the possibility for this is very low.

## Discussion

**Figure 8:**
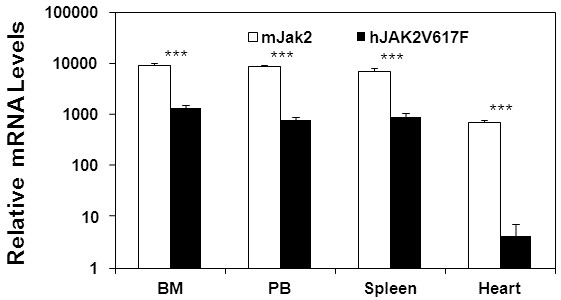
**Real time PCR assays revealed marginal expression of transgenic JAK2V617F in the mouse heart.** Expressions of the endogenous mouse Jak2 gene and the JAK2V617F transgene in the bone marrow (BM), peripheral blood (PB), spleen, and heart of 40-week-old JAK2V617F transgenic mice were determined by real time PCR. Data represent relative mRNA levels (mean ± SD, n = 3) normalized to mouse GAPDH in arbitrary units. *** P < 0.001.

By using JAK2V617F transgenic mice, this study demonstrates a direct association of JAK2V617F-induced MPN phenotypes with heart diseases. We believe that the later complication is a consequence of the former condition. This is supported by the fact that the development of MPN phenotype preceded the onset of cardiomegaly in these transgenic mice. In addition, the JAK2V617F transgene is controlled by the *vav-1* gene promoter which drives gene expression specifically in hematopoietic cells with minimal expression in the heart (see Figure 8 and ref. [19,26]). Therefore, JAK2V617F primarily affects the blood cells which in turn exert effects on cardiomyocytes and other cells in the heart through various physical and chemical interactions. Our study established a causal link between the MPN blood malignancy and heart diseases. This provides a mechanistic explanation for the high morbidity and mortality of MPN patients due to cardiovascular events [10-12]. JAK2V617F transgenic mice thus represent an excellent system to study cardiovascular diseases as well as blood disorders.

Cardiomegaly can usually be caused by coronary artery diseases or high blood pressure (hypertension). On one hand, we observed the occurrence of thrombosis in the canary artery of JAK2V617F transgenic mouse heart. This can presumably cause myocardial ischemia thereby leading to cardiac enlargement, and the increased platelets in the transgenic mice may contribute a higher incidence of thrombotic events. On the other hand, hypertension may represent a more general phenotype in the transgenic mice. High red blood cells in these mice directly increase blood viscosity and thus raise the peripheral resistance. It has been well documented that high red blood cells and hematocrit lead to hypertension [27-30]. In fact, hypertension and thrombosis are common initial presentation and complications with MPN patients. Our data suggest these patients may also develop cardiomegaly.

Our study revealed substantial accumulation of collagen fibers in the heart of JAK2V617F transgenic mice. This can contribute significantly to cardiac hypertrophy in addition to increased cardiomyocyte size. Cardiac fibrosis is considered to be the key to understanding heart disease. Recent research points to several key signaling pathways involved in cardiac fibrosis, providing biomarkers and potential targets for intervention. The JAK2V617F transgenic mice serve as a good *in vivo* system to dissect these pathways and identify growth factors and cytokines that trigger the activation of these pathways. Among the extracellular matrix in the heart, collagens are the most abundant fibrillar proteins [20,21]. Previous studies have mainly focused on type I collagen. Type I collagen can be detected by trichrome staining as shown in Figure 4, and it forms thick fibrils. However, type III collagen is also abundant in the heart but is seldom examined. Type III collagen forms so-called reticulin thin fibers. By using silver staining, we found a more profound change in the level of reticulin fibers in the extramyocyte space of hearts from JAK2V617F transgenic mice. Therefore, this method should be generally used to detect fibrosis in the heart. Reticulin fibers are also found in the liver, kidney and the spleen. Interestingly, JAK2V617F transgenic mice developed myelofibrosis in the bone marrow and spleen [1-3]. This is also recapitulated in JAK2V617F mice [19]. The myelofibrosis is mainly caused by accumulation of reticulin fibers. We believe that the reticulin fibers found in the heart of JAK2V617F transgenic mice may be caused by a similar mechanism by which myelofibrosis is regulated.

Inflammation has a major role in cardiac hypertrophy. Importantly, we observed infiltration of inflammatory cells in the muscle of hearts from JAK2V617F transgenic mice. This inflammatory reaction may be triggered by a cell necrosis due to myocardial infarction. Necrotic cells release soluble factors that cause migration of inflammatory cells and fibroblasts to the site of injury for repairing. This further leads to fibrosis as seen in this study (Figure 7). Interestingly, we also found mast cells among the infiltrated inflammatory cells. Mast cells have a major role in the pathophysiology of cardiovascular disorders [22]. Our data suggest that mast cells may promote fibrosis in the stressed and injured heart. We believe that the presence of JAK2V617F in these inflammatory cells further enhances their ability to infiltrate tissues thereby causing more profound damages. Identification of JAK2V617F has provided an excellent target for drug development. Many potent JAK2 inhibitors have been developed, and some have shown clinical benefits in treatment of myelofibrosis [31-34]. These inhibitors should also be useful for treating cardiovascular complications associated with MPNs.

## Conclusions

We have demonstrated that JAK2V617F-induced MPNs can further lead to cardiac hypertrophy in mice. This has major implications for our understanding of complications that occur in MPN patients. Our data help to explain the high morbidity and mortality of MPN patients due to cardiovascular events. We have further demonstrated that the cardiac remodeling in JAK2V617F transgenic mice is manifest in fibrosis involving not only type I collagen but also type III reticulin fibers. By linking a hematological malignancy with heart diseases, JAK2V617F transgenic mice thus represent a unique model system to study cardiovascular diseases as well as blood disorders.

## Materials and methods

### Animals

JAK2V617F transgenic mice were generated as previously described [19]. The JAK2V617F transgene is under the control of the *vav-1* promoter which drives gene expression only in hematopoietic cells. These mice have been crossed with wild type C57BL/6 mice for over 10 generations. Homozygous line A JAK2V617F mice were used in this study. These mice carry 26 copies of the JAK2V617F transgene [19]. Wild type C57BL/6 were purchased from The Jackson Laboratory and used as control together with non-transgenic siblings of JAK2V617F transgenic mice. At least 10 mice with about equal male and female representations were used for each control or experimental group. Animals were housed in ventilated cages under standard conditions. This study was carried out in strict accordance with the recommendations in the Guide for the Care and Use of Laboratory Animals of the National Institutes of Health.

### Mouse tissue collection, fixation, and sectioning

Mice were weighed and put under deep anesthesia through inhalation of isoflurane. Terminal blood collection was performed by removing the eyeball from the socket [35]. Hearts were then removed, blotted free of blood, and weighed. This is followed by fixation in 10% neutral buffered formalin overnight at room temperature and subsequent embedding in paraffin. Tissue sections (5 μm) were cut from various positions.

### Histochemical staining, image acquisition, and digital quantification

Tissue sections were deparaffinized and then subjected to H&E, Masson’s trichrome, reticulin, and toluidine blue staining by using reagents and kits from Sigma-Aldrich following standard protocols. Slides were viewed with an Olympus BX-51 upright microscope equipped with U Plan Fluorite objectives. Images were acquired using a DP71 digital camera with the DP-BSW-V3.1 camera control software (Olympus) and were processed with the Adobe Photoshop software. Quantification of histochemical stain images was done by using the NIH ImageJ program. Cardiac sections from at least 10 mice per group were analyzed. The thickness at the obtuse margin of the left ventricle of each heart was measured, excluding trabeculations. Cardiomyocyte size was assessed by measuring cross-sectional area of cardiomyocytes from H&E-stained fields randomly selected.

### Total RNA isolation and real time PCR analysis

Total RNAs were isolated from mouse tissues by using the RNeasy Mini kit (Qiagen), and single strand cDNAs were synthesized with equal amounts of total RNAs by using the QuantiTect reverse transcription kit from Qiagen. Real time PCR was performed with iQ SYBR Green Supermix (Bio-Rad) and primers specific for endogenous mouse Jak2, transgenic human JAK2V617F, and mouse glyceraldehyde-3-phosphate dehydrogenase (GAPDH) as previously described [19].

### Statistical analysis

Statistical analyses were performed using the GraphPad program. Differences of samples between 2 groups were accessed using *t* tests. *P* values less than 0.05 (2-tailed) were considered significantly different.

## Abbreviations

MPNs: Myeloproliferative neoplasms; PV: Polycythemia vera; ET: Essential thrombocythemia; PMF: Primary myelofibrosis; H&E: Hematoxylin and eosin.

## Competing interests

The authors declare no conflict of interests.

## Authors' contributions

KS performed the research and wrote the manuscript; WZ, YC, and WTH performed the research; PY and ZJZ designed the research and wrote the manuscript. All authors read and approved the manuscript.
